# Augmented Reality Framework to Measure and Analyze Eye–Hand Coordination in Stroke Patients with Unilateral Neglect: Proof-of-Concept Study

**DOI:** 10.2196/70985

**Published:** 2025-11-03

**Authors:** Jonathan Becker, Sophokles Ktistakis, Mirko Meboldt, Thomas Nyffeler, Dario Cazzoli, Quentin Lohmeyer

**Affiliations:** 1 ETH Zurich, Leonhardstrasse 21, Zurich, 8092, Switzerland, 41 44 632 26 1; 2 Neurocenter, Lucerne Cantonal Hospital, University Teaching and Research Hospital, University of Lucerne, Lucerne, Switzerland; 3 Faculty of Behavioural Sciences and Psychology, University of Lucerne, Lucerne, Switzerland

**Keywords:** visual neglect, stroke rehabilitation, augmented reality, eye-hand coordination, gaze anchoring, hand tracking, eye tracking, digital twin, motion capture, neurorehabilitation technology, unilateral spatial neglect, AR

## Abstract

**Background:**

Stroke is a leading cause of disability, often accompanied by unilateral spatial neglect (USN), which severely impairs recovery. Traditional assessments like paper-pencil tests provide limited insights into behaviors and eye–hand coordination during real-world tasks. Advances in hand pose estimation and eye tracking in combination with augmented reality (AR) offer potential for data-driven assessments of naturalistic interactions.

**Objective:**

This proof-of-concept study presents and evaluates a multimodal behavioral tracking system that captures gaze, body, and hand movements during interactions within an AR environment. Our primary goals are to (1) validate that this system can achieve robust and accurate interaction data capture in clinical settings, (2) show that the system can reliably detect known USN behavioral patterns, and (3) explore how comprehensive data can provide new understanding of eye–hand coordination deficits in USN.

**Methods:**

We developed an AR-based assessment system using Microsoft HoloLens 2 and an external body-tracking camera to capture real-time gaze, hand, and body movements in an interactive environment. Multimodal data streams were temporally synchronized, fused, and filtered to enhance spatial accuracy and availability. Tracking performance was benchmarked against a traditional optical motion-capture system to validate reliability. In a study, 7 patients with right-brain lesions with mild to moderate USN and 8 healthy controls participated. Each performed a designed reaching task, stamping virtual sheets of paper that appeared randomly on a table. We analyzed participants’ search behavior patterns to assess attentional biases and examined gaze anchoring timing during targeted reaching motions to explore potential eye–hand coordination deficits.

**Results:**

The fusion of hand-tracking data from the HoloLens 2 and external system reduced tracking loss from 25.7% to 2.4%, with an absolute trajectory error of 3.27 cm. The system demonstrated high usability and was well accepted by patients. Data from the control group confirmed the absence of intrinsic lateral biases in the system and task design. The USN group displayed typical search behavior through ipsilesional biases in gaze direction during visual exploration (median deviation 7.46 [1.61-9.48] deg, *P*<.05) and longer times to find contralesional targets (median difference 1.08 [0.20-1.80] s, *P*=.02). Additionally, the eye–hand coordination analysis revealed lateral differences in gaze anchoring during targeted reaching motions in the USN group, with earlier fixation on contralesional targets (median difference 112 [71-146] ms, *P*=.02).

**Conclusions:**

The proposed AR framework provides a novel, comprehensive data-driven method for capturing interaction behavior in a controlled, yet naturalistic environment. Our results demonstrated the system’s effectiveness in measuring hallmark USN symptoms, such as gaze and head orientation biases, and highlighted its potential to complement traditional assessments by offering deep insights into torso rotation and eye–hand coordination with a high resolution and accuracy. This data-driven approach shows promise for enhancing current USN assessment practices and gaining new insights into patients’ behaviors.

## Introduction

Stroke represents a profound public health challenge, affecting one in four adults during their lifetimes [1]. Of the numerous poststroke impairments, unilateral spatial neglect (USN) is a particularly significant barrier to patient recovery: Evident in up to 43% of patients after a right-hemispherical brain lesion (RBL) [2], this condition is characterized by a reduced ability to attend to contralesional stimuli, impacting essential daily activities [3,4]. Consequently, the rehabilitation journey is fraught with challenges, including heightened fall risks, poorer rehabilitation outcomes, extended hospitalization time, and reduced likelihood of being discharged home [5-7].

In 75% of USN cases, patients present with egocentric visual USN [8], marked by a bias of visual exploration through head and eye movements toward the ipsilesional side [9]. However, USN is a heterogeneous condition, encompassing a spectrum of attentional deficits across visual, tactile, auditory, and proprioceptive domains [10-12]. Consequently, multiple tests are typically employed to measure the severity of USN. Paper-pencil tests such as the Bells/Stars Cancelation [13] and Line Bisection [14] are commonly used due to their good sensitivity among paper-pencil assessments [15]. However, discrepancies between test results and patients’ real-world behavior are frequently reported [16].

Behavioral assessments like the Catherine Bergego Scale address this gap by evaluating neglect during several activities of daily life, but they require labor-intensive direct observation and offer limited quantitative insight into task execution [17]. Technological approaches, such as gaze analysis during free visual exploration, provide quantitative insights but are restricted to specific test settings [18]. Evidence suggests that attentional biases in USN extend beyond eye movements, being influenced by motor acts and proprioceptive feedback. For instance, the orientation of the trunk mid-sagittal plane was shown to modulate the perceived forward direction and shift neglected space [19-22]. Moreover, proprioceptive cues, such as pointing, guide the direction of visual attention [23]. Accordingly, patients with USN exhibited biases of visual attention toward the ipsilesional sides of these cues [24].

These findings highlight the need for a comprehensive behavioral tracking system that can capture eye, body, and hand movements during task execution in environments that closely mimic real-world conditions.

Virtual reality (VR) or augmented reality (AR) systems that are able to track gaze and hand movements partially address these requirements. VR has been explored to simulate real-world interactions for studying USN [25]. However, immersive VR introduces new challenges such as user disconnection from the real environment, proprioceptive disconnection (eg, the inability to see one’s own hands), and motion sickness [26]. By contrast, AR overlays simulated elements onto the real world, creating a mixed reality experience, addressing aforementioned discomforts [26] and integrating both physical and virtual objects into experiments. Consequently, AR has recently seen a growing application in neuroscience and rehabilitation [27,28].

However, despite these advantages, standalone AR systems still fall short of the comprehensive behavioral tracking requirements identified earlier. The inside-out tracking approach of typical AR systems like the Microsoft HoloLens 2 (where sensors on the headset track the environment) provides limited full-body tracking beyond hands and head position. Additionally, hand tracking can fail when users’ hands are not positioned directly in front of them or during complex object interactions.

To address this gap, researchers have begun complementing inside-out tracking with external motion capture (Mocap) systems [29,30]. In clinical settings, external tracking systems combined with AR have primarily been used to position virtual objects relative to physical ones, particularly for surgical guidance applications [31,32]. However, there is limited evidence of this combination being utilized to capture comprehensive behavioral data in clinical rehabilitation contexts [33].

Accordingly, we propose a novel framework encompassing a head-mounted AR system (HoloLens 2, Microsoft USA [34]) and a single RGB-D camera (ZED 2i, Stereolabs USA), providing the following:

- A controlled environment allowing for a wide range of user interactions with both digital and real-world elements.
- Accurate tracking of gaze orientation, hand movements, and body posture during task execution, with improved hand-tracking accuracy and availability enabled by sensor fusion.
- Detailed digital reconstruction of interactions to enable comprehensive behavioral analysis.
- Automated data integration and metric extraction to enable quantitative analysis, with a focus on eye–hand coordination.

The goal of this proof-of-concept study is to demonstrate the feasibility and utility of our tracking framework through a systematic validation approach. Our validation encompasses both a technical accuracy assessment against a marker-based VICON system and clinical validation through the detection of known USN symptoms. Beyond confirming that the framework captures established USN behavioral patterns, we explore lateral differences in eye–hand coordination during goal-directed actions as a novel behavioral marker. With this, our framework shows promise in enhancing current USN assessment practices and gaining new insights into patients’ behaviors.

## Methods

### System Overview

**Figure 1.**
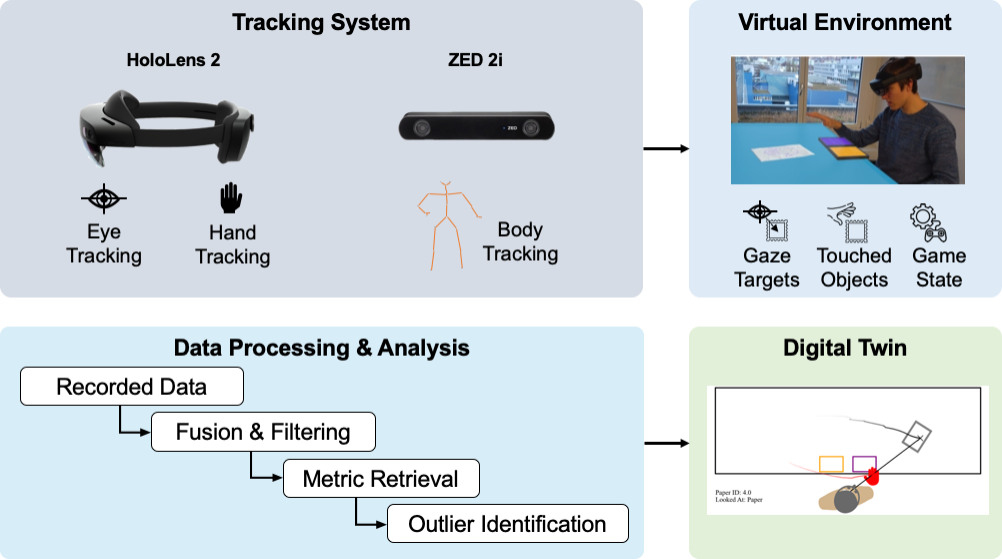
Overview of the system.

The system (Figure 1) comprised three main parts. (1) A tracking system, which measures and records gaze direction, hand, and body movements. It enables interactions with (2) a simulated virtual environment, which is overlaid onto the real world using a head-mounted AR display. (3) An offline data-processing and data-reconstruction system creates a digital twin of the captured interactions, allowing for in-depth analysis.

### Tracking System

We obtained interaction data from multiple sources. The HoloLens 2 provided gaze and hand-tracking data through the Mixed Reality Toolkit API [35]. These data were recorded alongside the state of the virtual environment, at a frequency of 40 Hz, capturing the users’ view at each moment. Additionally, an external RGB-D camera (ZED 2i, Stereolabs, USA) was set up in front of the users, facing them.

The coordinate frame was aligned to that of the HoloLens during setup by taking a picture of a checkerboard with both cameras and extracting the relative transformation between the two. Using the manufacturer-provided body-tracking algorithms [36], body key points were extracted, including hand and shoulder joint positions. This pose information was recorded at a frequency of 30 Hz. The hand-tracking data were additionally streamed in real time to the HoloLens to complement the on-device hand tracking that was at times subject to tracking loss.

### Virtual Environment Design

**Figure 2.**
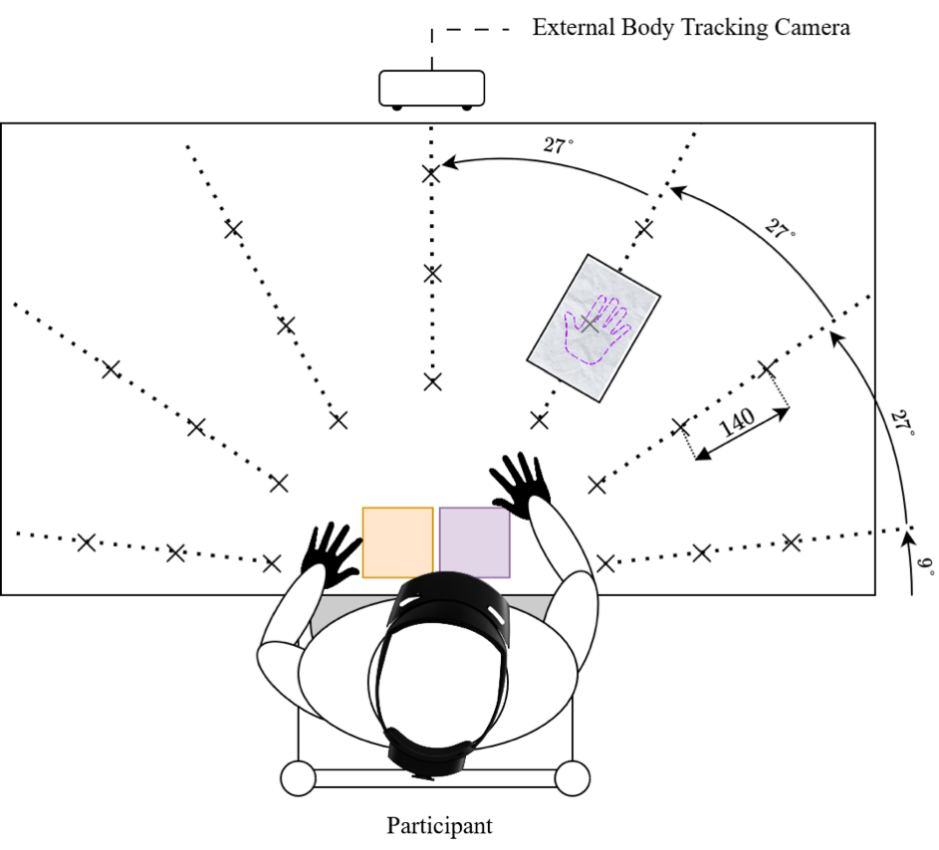
Top-down view of the game setup. Virtual sheets of paper appear in 21 possible locations indicated by X, radially distributed and spaced at 140 mm by default. Two virtual stamp pads are always shown in front of the user. The ZED camera for external body tracking is set up opposite of the user with a view of the entire table and the user’s pose. Image of HoloLens 2 from [34].

The virtual environment was designed to assess the hand- and eye-tracking capabilities of our system, specifically its ability to capture metrics relevant to USN. To encourage naturalistic behavior, it was structured as a game around a familiar, easy-to-learn task that required repeated, precise reaching motions: stamping virtual sheets of paper. For this task, users sat in front of a physical table overlaid with a holographic table. Two virtual ink pads, one orange and one violet, were shown to participants, positioned as shown in Figure 2. During the task, virtual sheets of paper appeared sequentially in a random order across 21 locations distributed radially (Figure 2). Each sheet displayed a hand outline, color-coded in orange or violet. The users selected the color by putting their hand into the corresponding virtual ink pad in front of them. They then performed a reaching motion to stamp the paper, after which it disappeared, and a new one appeared. Participants were told that their main goal was to perform this task as fast as possible, but to ensure goal-oriented reaching and precise movements toward the targets, they were also told to stamp the papers as precisely as possible within the respective hand outline.

To promote naturalistic behavior while ensuring data comparability, implicit constraints were implemented. A brief, randomized delay of 1 to 3 s after each paper was stamped encouraged users to reset their posture between trials. Moreover, the virtual sheets never appeared adjacent to each other, that is, as direct neighbors in any direction, to minimize carryover effects from the previous location. By requiring ink selection prior to stamping, each movement began from a central position and interactions followed a consistent sequence: locating the paper, selecting the ink, and finally stamping. These constraints ensured the independence and comparability of each episode without explicitly directing user actions.

An initial tutorial was introduced to help participants understand the game and the interaction with the system. This allowed users to familiarize themselves with the task and the limited field of view of the HoloLens 2, which required them to turn their heads to see all possible paper positions on the table. To ensure that all sheets were comfortably reachable, the experimenter could adjust distances between papers during the tutorial so that even the farthest sheet remained within arm’s reach. This setting was kept constant during the experiment.

The repeatability of each experiment was ensured by fixing the seeds on the random number generators. Moreover, potential biases were avoided by balancing out the color of the outline on the paper, ensuring that both colors occurred equally often within and between the left- and right-hand sides of the user.

The stamping task allowed users to interact with a physical surface that aligned with the virtual one, providing direct haptic feedback. This design choice was informed by insights from pilot experiments and previous work within our research group, highlighting the importance of tactile interaction [37].

To enhance engagement, a non-directional “bling” sound, similar to a phone notification, signaled the appearance of each new paper, while a chime rewarded users when they correctly stamped a sheet. Additionally, if a user could not find the paper within ten seconds, the system automatically advanced, causing the current sheet to disappear and the next one to appear.

### Data Processing

For comprehensive behavioral analysis, accurate hand movement, gaze, and body position tracking were required. To address temporal and spatial misalignment, sensor noise, and data loss, the recorded data underwent extensive processing outlined in this section.

Temporal misalignment between the external tracking and the HoloLens 2 was corrected in two steps. During the recording, the hand poses were streamed to the HoloLens. An initial time offset estimate was obtained from the difference in sender and receiver timestamps. However, this still included network and processing delays and an error introduced by the difference in sampling rate (40 Hz vs 30 Hz). The time offset was therefore refined by maximizing cross-correlation of the normalized hand positions from both sources. Across all recordings, the cross-correlation of hand poses was on average 0.96, with external tracking data arriving with an average delay of 92 ms.

To address tracking losses and noise from either source, hand-tracking data from both the HoloLens 2 and external system were fused using a Kalman filter with a standard constant acceleration motion model. The two coordinate systems were initially aligned using a checkerboard calibration, but residual spatial errors were addressed by estimating a 3D offset within the filter. To avoid introducing temporal delays, Kalman smoothing was applied with a backward pass over the time series to achieve zero-phase filtering [38]. The filter parameters were tuned based on expected hand motion characteristics and system accuracies (Table 1). Innovation gating was used to reject outlier measurements that fell outside the 90% confidence region based on the calculated Mahalanobis distance.

**Table 1.** Kalman filter parameters.

Parameter	Value	Description
Process noise (acceleration)	3 m/s²	Expected SD of hand acceleration
Process noise (spatial offset)	0.0001 m/s	Random walk SD for coordinate alignment
External tracking measurement noise	0.02 m	SD of external tracking poses
HoloLens measurement noise	0.01 m	SD of HoloLens poses
Innovation gate threshold	90%	Confidence region for outlier rejection

Gaze data was recorded in two ways: On one hand, the gaze direction and origin were recorded, from which the construction of a gaze ray was possible. On the other hand, the intersections of the gaze ray with objects in the scene, for example, “Paper, Table, Ink Pad,” were computed at each update, and the target of the gaze was recorded. Given the reported variance of 1.5° to 3° in HoloLens 2 gaze directions [39], temporal filtering was applied to both time series. For the gaze target-object filtering, only Papers and Ink Pads were considered as targets of interest. A median filter over seven samples (spanning 0.175 s at 40 Hz) was applied to the time series. As a result, a target was considered fixated when first intersected, if subsequently the gaze remained on it for at least 50% of the sliding window duration (~0.085 s). The gaze origin and gaze direction vectors were filtered with a median filter with a 0.35 s window.

Head poses were obtained directly from the HoloLens 2 without filtering, as it exhibited little noise due to the device’s built-in robust localization system.

The torso rotation was reconstructed from the tracked chest, left, and right shoulder joints of the external tracking data. Outliers were removed based on sanity checks, asserting that the head position was always between the two tracked shoulder joints.

### Metric Extraction and Data Visualization

To validate the system’s capability to measure common neglect symptoms, we focused on metrics that evaluate search behavior and attentional shifts therein. Our analysis incorporated spatial orientation metrics during the search phase (from paper appearance until target identification): average gaze direction, head rotation, and torso rotation, all computed relative to the table coordinate system using filtered sensor data and estimated forward vectors from the processed data.

For eye–hand coordination analysis, we examined the temporal relationship between gaze anchoring and motion onset. While gaze timing could be directly extracted from the gaze–target time series, determining precise motion onset required a more sophisticated approach.

An initial estimate, given by the recorded time the hand left the ink pad, was refined by identifying the optimal velocity minimum from candidate minima within the movement window. The selection was based on a multifactor scoring system that weighted (1) spatial proximity to the ink pad position, (2) hand velocity magnitude, (3) directional alignment with the target movement vector, and (4) temporal consistency with stamping events. The candidate with the lowest combined score was selected as the movement onset point.

Despite this sophisticated approach, various technical and environmental factors could still affect motion onset detection accuracy, creating the need for a systematic data quality review. To address this, we implemented an automated outlier detection system that flagged an episode for review in case the duration of the motion exceeded 1.5 times the interquartile range (IQR) beyond the upper or lower quartile for individual user measurements (accrued metric over all episodes for that user).

Other metrics, such as the time it took to find a paper after its first appearance, were also processed by this system to detect anomalies resulting from technical issues such as occasional failures to register fixations or interactions.

**Figure 3.**
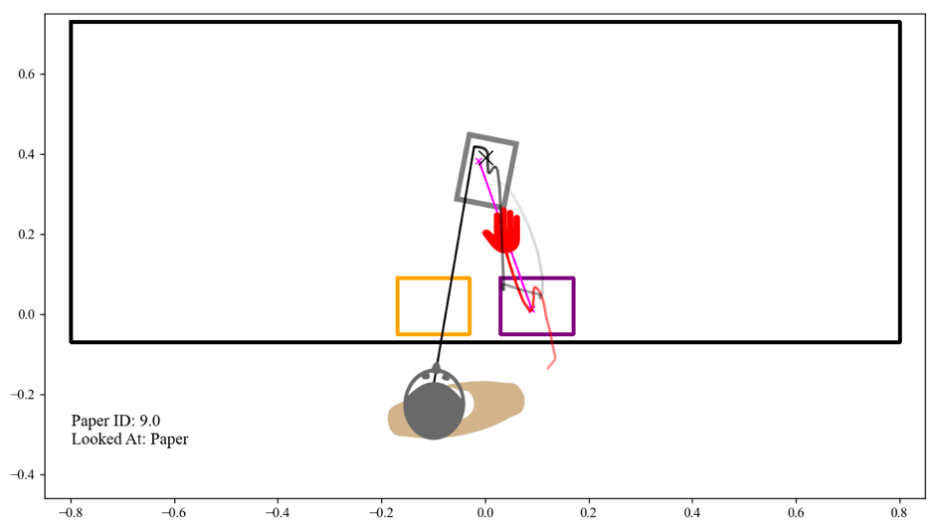
Snapshot of digital twin replay of example data from our dataset. A black X marks the current fixation point. The magenta line connects the current motion start and end point. The torso with estimated orientation is shown in brown, together with the tracked positions of the hand and head. A red path shows the hand’s position history, and a black path shows the user’s gaze target over the past 3 seconds, both fading with time.

To facilitate the review of these episodes and, in a broader sense, enable the qualitative analysis of the recorded interactions, we implemented a digital twin: an interactive 2D visualization tool reconstructing user interactions (Figure 3). This digital twin provided an animated top-down view of the simulated environment, displaying the users’ head, torso, and hand poses, gaze directions, and hand movement data. This allowed for a visual review of the flagged episodes to determine if the anomalies resulted from technical errors rather than natural human behavior. Any episodes where the visualization revealed tracking loss during critical phases, fixation or interaction registration failures, or other clear technical errors that compromised the affected metrics were marked as outliers. These episodes were excluded from the subsequent analysis.

The digital twin tool abstracted sensitive information, enabling secure and collaborative qualitative analysis, which both clinicians and researchers found valuable for reviewing patient behavior. Its visualization of extracted metrics provided an intuitive way to replay and contextualize gaze, motion, and compensatory strategies, making it a practical aid for identifying patterns and generating insights to guide research and rehabilitation approaches.

### Tracking Accuracy Evaluation

To evaluate the hand-tracking accuracy of the HoloLens 2, the ZED 2i body tracking, and the fused hand trajectories, we compared their 3D hand positions against ground-truth data recorded at 200 Hz with a VICON motion capture system.

Participants were equipped with infrared markers placed on both shoulders, the chest, and the index knuckle of each hand. Temporal alignment of the data was achieved by maximizing cross-correlation across speed and acceleration of hand movements. All signals were resampled to a common temporal grid. The spatial alignment was initialized by finding the rigid transformation that aligned the tracked shoulder and neck positions between the systems. It was then refined via an Iterative Closest Point (ICP)-like approach, maximizing global alignment of the hand positions between the Mocap system and the filtered hand positions.

### Study Design

A proof-of-concept study was conducted with patients experiencing neglect symptoms after an RBL. Each patient played three rounds of the “game,” where one round consisted of one paper appearing at each of the 21 locations, for a total of 63 episodes. The first round of the game was only played with the right hand; the subsequent two rounds presented the patients with papers containing left- and right-hand outlines, requiring patients to select the correct hand for the interaction. The order in which the papers appeared, as well as their color and hand outline, was randomized across games. The randomization was balanced to obtain two right-handed interactions per paper location for a total of 42 episodes (21 episodes in the first game, 12 in the second game, and 9 in the last game). The color randomization was equally balanced, resulting in a total of 33 episodes showing purple papers and 30 episodes with orange ones.

Before the experiment was started, the participants were asked to calibrate the eye tracker of the HoloLens 2 using Microsoft’s calibration tool. To complete the calibration, a Vuforia marker [40] was used to align the holographic table with the physical table.

The patients were subsequently presented with a tutorial phase, during which no data were recorded, and the sheets did not disappear. The positions of the sheets in the tutorial were the same as in the actual game, but the sequence and colors were different, to avoid learning effects. During this stage, the experimenter assisted patients in locating the sheets by referring to the external application monitor, which displayed the current sheet’s position.

Once a participant had independently located and stamped six consecutive papers, the tutorial was terminated, and the actual assessment began. They were informed that the time was being recorded and instructed to perform the stamping task as fast and as accurately as possible.

While it was initially intended to also consider left-handed motions, we soon found that many of the patients had impairments to the contralesional limbs, which could not be attributed to USN. In the results, we therefore only considered the motion data from the right-handed interactions. For perception-only metrics, like the time until a paper was first looked at, all episodes were included in the analysis.

### Ethical Considerations

The study was conducted in accordance with the latest version of the Declaration of Helsinki and received approval by the Ethics Committee of the Canton of Lucerne (ID 2017‐02195). Before participation, all participants were informed about the content of the study, the steps and procedure of the experiment, and the goal of the study. Participants provided written informed consent and were explicitly informed of their right to revoke their consent at any time throughout the study or later. All data were anonymized to protect participants’ privacy and confidentiality. Participants did not receive any compensation for their participation.

### Usability

At the end of the experiment, the participants completed a usability questionnaire consisting of the following statements, each rated on a 5-point Likert scale where 1 was annotated with “strongly disagree” and 5 with “strongly agree (translated from German):

- Interacting with the elements (eg, paper, stamp) was comfortable
- I intuitively understood how to interact with the elements (paper, stamp).
- The game instructions were clear and easy to understand.
- The game maintained my attention from beginning to end.
- I enjoyed playing the game.
- The challenges in the game were appropriate and well-balanced.
- I felt overwhelmed while playing the game.
- The game had a good pace—neither too slow nor too fast.
- I felt stressed by the game.

## Results

### Patient Characteristics

The study was conducted with seven right-handed patients (3 women and 4 men), between 49 and 83 years old (median 75 [58-78] y), who had an RBL. The participants were diagnosed with mild to moderate USN based on clinically relevant scores in a battery of tests (Table 2) consisting of mean gaze position (MGP) during free visual exploration (FVE) [18], bell’s cancelation tests [13], and behavioral analysis using the Catherine Bergego Scale score [4,17]. While interactions with both hands were recorded, our analysis focused on the dominant and ipsilesional right hand. Only patients with little to no impairment of their right hand and arm movement were selected, which was assessed using the LIMOS score [41]. Due to time constraints, two patients (P1 and P5) completed only two out of three recordings during the experiment duration. For patient P5, this was anticipated, and they were asked to play the game with their right hand twice.

**Table 2.** USN group individual test scores on relevant clinical tests performed by clinicians prior to the study.

Patient ID	Gender	Age (y)	CBS^a^	MGP FVE^b^	CoC^c^	LIMOS^d^ right hand and arm movement
P1	Female	83	4	3.92	0.04	4/5
P2	Male	49	1	1.45	0.02	5/5
P3	Male	79	0	0.43	0.15	4/5
P4	Female	77	7	3.25	0.37	5/5
P5	Male	67	5	1.33	0.09	5/5
P6	Female	74	12	2.52	0.11	5/5
P7	Male	49	0	2.00	0.09	4/5

a CBS: Catherine Bergego Scale (0%‐30%, higher=more neglect).

b MGP FVE: Mean gaze position during free exploration (cut-off>1.33°, rightward=positive).

c CoC: Center of cancellation (cutoff>0.081, left neglect=positive).

d LIMOS: 4=slowed and 5=independent.

The control group consisted of eight healthy individuals (1 female and 7 males) between 20 and 60 years old (median 31 [23-51] y). While none of the participants in the USN group had previous experience with AR devices, four out of the eight participants in the control group had previously used the technology.

### Accuracy and Availability of Hand Tracking and Torso Rotation

Hand-tracking accuracy was evaluated for the HoloLens 2 and ZED 2i tracking system individually, as well as for their fused and filtered data, using the VICON Mocap system as ground truth. For evaluation, three male users independent from the clinical study participants were chosen, and measurements were obtained from gameplay with either hand, resulting in a total of 6 recordings (3 left hand and 3 right hand). We report the tracking accuracy during the relevant dynamic reaching motion only. The results in Table 3 show that the fused approach and the HoloLens 2 standalone achieved similar hand-tracking accuracies, with root mean square errors (RMSE) of 3.27 cm and 3.54 cm, respectively. The ZED 2i data contained large outliers (52.5 cm at the 95th percentile), which increased the RMSE to 37.1 cm. The median absolute error (AE) was also larger at 5.51 cm. The HoloLens 2 had frequent tracking loss, resulting in 74.3% availability. The ZED 2i and the fusion of the two sources achieved much better rates of 97.2% and 97.6%, respectively.

**Table 3.** Tracking accuracy results versus VICON data.

Tracking method	RMSE^a^ (cm)	Median AE^b^ (cm)	95th percentile AE^b^ (cm)	Availability^c^
HoloLens 2	3.54	2.71	6.30	74.3%
ZED 2i	37.1	5.51	52.5	97.2%
Fusion	3.27	2.24	5.80	97.6%

a RMSE represents the root mean square average trajectory error during the reaching motion.

b Median AE and 95th percentile AE show the distribution of absolute errors across all samples.

c Availability indicates the percentage of frames with valid tracking data.

The torso rotation extracted from the ZED2i body tracking was compared to that obtained from the VICON system and evaluated during the search phase. The median signed error was 0.26°, with a median AE of 1.45° and 97.1% availability (Table 4).

**Table 4.** Torso rotation accuracy and availability against VICON data during free visual exploration^a^.

Metric	Value
Median angle error (signed)	0.26°
Median absolute angle error	1.45°
95th percentile absolute angle error	4.41°
Availability	97.1%

a Availability indicates the percentage of frames with valid tracking data.

### Omissions

While patients received assistance and cues to search the entire space during the tutorial, no cues were provided during the actual experiment to avoid influencing search behavior. Papers that remained unfound after 10 s automatically disappeared, and the next paper appeared to maintain game progression. This time limit resulted in some omissions in the USN group: 9.3% (15/161 episodes) of papers on the left side and 3.3% (5/151 episodes) on the right side were not found within the time limit. The control group showed no omissions, nor were any omissions recorded for centrally positioned papers in either group.

### Biases During Search Behavior

In line with previous research [8,9], the visual exploration of patients with USN was hypothesized to be biased toward the ipsilesional side. Using the proposed system, we analyzed the gaze direction during the search phase, as well as torso and head rotation. In the following, all statistical tests were performed using a Wilcoxon signed-rank test (due to the small number of participants) for a zero-mean hypothesis with a two-sided alternative. The level of significance was chosen at *α*=.05.

**Figure 4.**
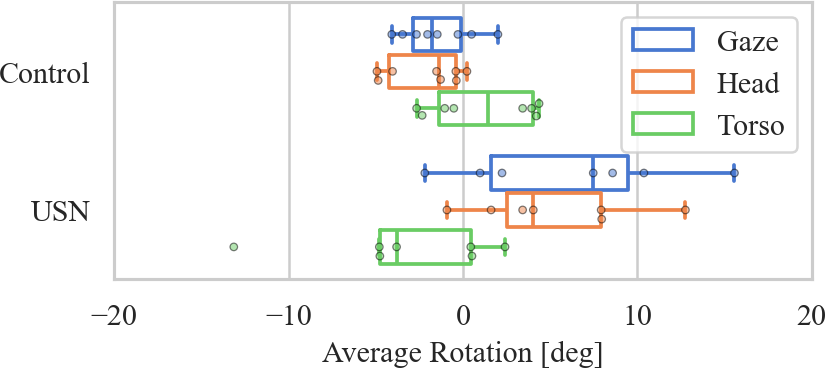
Boxplots and data points for average orientation of gaze, head, and torso relative to the table in front of the users. Boxes represent the IQR, the solid lines indicate the median, the whiskers indicate the minimum and maximum values that are not considered outliers.

The results in Figure 4 show that the USN cohort exhibited a significant ipsilesional shift in the average gaze direction during FVE (median 7.46 [1.61-9.48] deg, *P*=.05). Similarly, a significant rightward trend of their average head rotation was observed (median 4.03 [2.52-7.94] deg, *P*=.03). A trend of an average torso rotation toward the contralesional side in the USN group did not achieve statistical significance (median −3.81 [−4.78 to 0.48] deg, *P*=.22) . In the control group, the average head orientation was shifted slightly, but significantly, toward the left during the search phase (median 1.40 [−4.25 to −0.04] deg , *P*=.02). Aside from that, the zero-mean hypothesis could not be rejected for the average gaze direction (median −1.75 [−2.87 to -0.10] deg, *P*=.11) and the average torso rotation (median 1.44 [−1.37 to 4.00] deg, *P*=.31) in the control group.

To investigate the effect of biased search behavior on the time it took users to find sheets of paper placed on the left and right sides of the table, we conducted a pairwise comparison. Each sheet on the left (within the two leftmost radial sectors) was matched with its mirrored counterpart on the right, and for each, we calculated the average time it took a user to find it across multiple trials. We then compared the average time for each paper on the left to the average time for its counterpart on the right. For example, if a user took an average of 8 s to find a specific sheet on the left and an average of 5 s to find its counterpart on the right, the difference for that pair would be +3 s.

This difference was computed for each pair and then averaged across all pairs per user to yield an overall measure of lateral difference. In the control group, out of the total 504 recorded episodes, 64 were flagged and removed as outliers (12.7%), whereas 50 of the total 399 episodes were removed in the USN group (12.5%). Any pairs with no data on either side were excluded from the analysis.

**Figure 5.**
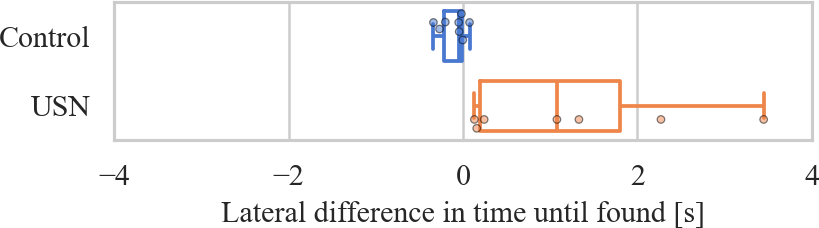
Pairwise differences of the time until each paper was found on the left and right side. A positive difference indicates that the papers on the right were found sooner. Box represents the IQR, the solid line indicates the median, the whiskers indicate the minimum and maximum values that are not considered outliers*.* Control: n=8*,* median −0.05 s [−0.22 to −0.01]*, P*=.08; USN: n=7; median 1.08 s [0.20-1.80], *P*=.02.

The results (Figure 5) indicate that the USN group took significantly longer to find papers on the left side compared to the right (median difference 1.08 s [0.20-1.80], *P*=.02), whereas no significant difference in the control group was observed.

### Differences in Timing of Gaze Anchoring

As an important aspect of eye–hand coordination, we investigate gaze anchoring behavior during targeted motion. A typical episode involved the following steps: (1) the participants were located and fixated on the paper; (2) gaze was shifted to the ink pad while the participant selected a color with their hand; (3) hand motion was initiated away from the ink pad toward the remembered location of the paper; and (4) gaze anchoring was shifted back to the paper to guide the ongoing motion.

Eye–hand coordination is a complex field of research. One aspect that is often considered is the timing of fixations with respect to intent and concrete actions [37]. In patients with USN, disrupted spatial attention may particularly affect this temporal coordination between gaze and movement. In the following, we therefore analyzed lateral time differences between (3) and (4). This was done using a pairwise comparison, similar to the previous section. For each side, we calculated the average time between the onset of hand movement and the fixation of the target paper, comparing these times between left- and right-hand sides. For example, if a user fixated on a sheet 100 ms after initiating hand movement toward it on the left side and 300 ms on the right, the difference for this pair would be −200 ms. The outlier review resulted in 52 of the 336 episodes with right-handed interaction data (15.5%) being rejected in the control cohort and 47 out of 246 (19.1%) in the USN group.

**Figure 6.**
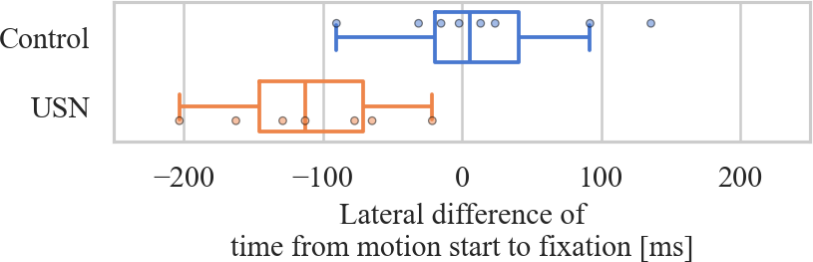
Pairwise difference between the sheets on the left and on the right side and of the time between motion onset and fixation of the motion target. A negative difference indicates that sheets on the left were fixated earlier. The box represents the IQR, the solid line indicates the median, and the whiskers indicate the minimum and maximum values that are not considered outliers*.* Control: n=8, median 5 ms [-19 to 41], *P*=.74; USN: n=7, median −112 ms [-146 to -71], *P*=.02*.*

The results (Figure 6) show that the USN group fixated on the target of their motion significantly earlier for targets on their left compared to their right (median difference of 112 ms [-146 to -71*], P*=.02). In contrast, the control showed no significant lateral difference (*P*=.74).

Given that gaze anchoring has been shown to shift in time with longer duration movements [42], we examined whether the observed laterality in gaze timing could be explained by corresponding differences in movement durations. To assess this, we analyzed the relationship between lateral differences in gaze timing and lateral differences in the duration of reaching motions across participants. Pearson correlation analysis revealed a moderate correlation, not achieving statistical significance (control: *r*=.20, *P*=.63; USN: *r*=.67, *P*=.10). Additionally, we examined whether patients with USN showed systematic differences in motion duration between left and right sides. Despite a mean difference of −80 ms, the median difference was effectively zero (0.8 [-204 to 63] ms), and a zero mean hypothesis could not be rejected (*P*=.58).

### Usability

Table 5 shows the median ratings for all usability questions. Patients rated the game as comfortable (median 4) and intuitive (median 5), with high scores for clear instructions (median 5), enjoyable gameplay (median 5), and balanced challenges (median 4). Participants generally felt the game was engaging (median 4) and appropriately paced (median 4), with minimal reports of stress (median 1) or overwhelm (median 1).

**Table 5.** Overview of the usability study questions and answers.

Question	Median
Interacting with the elements (eg, paper, stamp) was comfortable.	4
I intuitively understood how to interact with the elements (paper, stamp).	5
The game instructions were clear and easy to understand.	5
The game maintained my attention from beginning to end.	4
I enjoyed playing the game.	5
The challenges in the game were appropriate and well-balanced.	4
I felt overwhelmed while playing the game.	1
I was motivated to continue playing.	4
The game had a good pace—neither too slow nor too fast.	4
I felt stressed by the game.	1

## Discussion

### Accuracy and Availability of Hand Tracking and Torso Rotation

The HoloLens standalone method clearly demonstrates the tracking loss issue described in the Introduction. The data from the ZED 2i, while achieving excellent availability, suffered from both higher systematic errors, likely due to coordinate system misalignment, and significant noise, resulting in poor overall accuracy. Our fusion approach combined advantages of both sources, achieving better tracking accuracy than the HoloLens 2 while maintaining near-complete availability.

Torso angle tracking achieved high availability with no systematic bias, as evidenced by the small median signed angle error (0.26°). However, the median absolute angle error of 1.45° is relatively large compared to the clinical effects found during the search behavior phase, limiting the confidence in these results. This could possibly be improved in the future by ensuring better alignment between the external tracking system and the HoloLens 2 coordinate system.

### Biased Search Behavior in Patients With USN

The analysis of the MGP during the search phase revealed, as expected, an ipsilesional shift in the patients with USN with respect to the table center. This was consistent with previously recorded behavior of patients with USN [43-45]. The shifted MGP also explains the prolonged time until papers on the contralesional side were found.

A significant feature of our method compared to traditional eye tracking during FVE is that it allows for free rotation of the head and torso. Analyzing the search behavior with this approach revealed that patients exhibited a bias of their head rotation toward the ipsilesional side, aligning with their gaze direction. Moreover, some patients rotated their trunks slightly toward the contralesional side, although this was not a behavior that achieved significance among the USN group. Nonetheless, this prompts further investigation into the observed behavior, as trunk orientation is known to define the reference frame for visual neglect. Hence, a rotation toward the contralesional side could have reduced the attentional bias to a certain degree in those patients, as reported by [19,20,46]. In general, these findings emphasize the importance of measuring torso rotation for a comprehensive assessment of USN symptoms.

The control group exhibited a slight leftward bias of their head rotation relative to the table, which can be explained by the intrinsic left-to-right scanning tendency, sometimes associated with reading direction, and right-hemispheric dominance for attention prevalent among healthy individuals [47,48].

### Lateral Differences in Timing of Gaze Anchoring

The observed difference in gaze anchoring timing between the left and right sides encourages the hypothesis that neglect may impact eye–hand coordination in the USN group, though this remains a preliminary finding. In both groups, users generally initiated the motion from the ink pad to the paper “blindly,” relying initially on the remembered position of the paper. However, in the USN group, the results indicate that gaze anchoring occurs earlier in the motion for targets on the left compared to the right.

Several factors may contribute to this asymmetry. First, the USN group might have put less trust in their memorized target positions on the left side, prompting them to fixate on those targets sooner. Supporting this theory, previous works have reported increased localization errors on the contralesional side [49]. According to [50], the visuospatial working memory was negatively affected after patients shifted their attention toward stimuli ipsilesional to the memorized location. In our experiment setup, such a stimulus was present, as the stamping task involved an attentional shift back to the centrally positioned ink pads, requiring patients to memorize a contralesional location. Additionally, reaching with the right hand toward a target on the left may also have induced an intermediate attentional shift toward the ipsilesional side, reinforcing this effect.

Second, differences in movement duration could partially account for the gaze anchoring asymmetry [42]. We observed a moderate correlation between lateral differences in gaze timing and movement in the USN group (*r*=.67, *P*=.10). However, the movement duration differences showed high variability and no consistent lateral pattern. This suggests that those differences cannot fully explain the gaze timing asymmetry. Given the small sample size, further investigation with a larger cohort is needed to clarify this potential contribution.

### Usability

The positive feedback across several aspects of the framework and game suggests that it was accessible and motivating for users, supporting the feasibility of using it for more extensive clinical trials and during rehabilitation with patients with USN.

### Limitations

First, the lack of a stroke-only control group (without USN) limits our ability to confidently attribute the observed effects specifically to USN. Additionally, substantial demographic disparities existed between groups, which are known to influence motor performance and limit the comparability of motor-related metrics between groups. Future studies should include a stroke control group without USN, matched on demographics, to better isolate USN-specific effects.

Second, both groups had relatively small sample sizes, with only seven patients in the USN group whose neglect severity varied from mild to moderate. This might have inflated the large effect size (Cohen *d*≈2.3) of the lateral difference in gaze-anchoring timing in the USN group. Replication with a larger cohort may be necessary to validate these findings.

Third, measurement precision of torso rotation was comparatively low and limits the confidence in the associated results. Fourth, to obtain the tracking accuracy results, the VICON data had to be spatially and temporally aligned to the HoloLens in postprocessing. Therefore, these results reflect tracking accuracy within the HoloLens’ coordinate system rather than absolute world coordinates.

Despite these limitations, the results align with the hypothesized behavior of patients with USN, strengthening our confidence in the validity of the results.

### Conclusions

In this work, we developed and validated a comprehensive behavioral tracking system for patients with stroke and USN that can capture eye, body, and hand movements during task execution in a mixed reality environment. By integrating a head-mounted AR display with an external body tracking system, we obtained naturalistic, multimodal interaction data in patients with stroke and USN. The system combines a game-like task with automated metric extraction and data visualization, offering a scalable tool for quantitative and qualitative behavioral assessment.

Importantly, the system demonstrated a significant improvement in hand-tracking accuracy and availability over the standalone hand tracking of the HoloLens 2 or ZED 2i, with a fused hand-tracking error of 3.27 cm RMSE at 2.4% tracking loss.

In a proof-of-concept study with seven RBL patients with mild to moderate USN, the framework captured behavior consistent with known USN symptoms, such as ipsilesional gaze biases and delayed contralesional target detection.

The naturalistic task design combined with our system’s multimodal data capture revealed new insights into USN visuomotor behavior. In particular, we discovered a pattern where patients directed their gaze toward movement targets earlier when those targets appeared on their contralesional side compared to their ipsilesional side.

This framework opens several promising research and clinical avenues. Larger patient cohorts and appropriate control groups could provide more robust quantitative insights into USN-related visuomotor deficits. Future investigations of nondominant hand use and bimanual coordination, and more extensive movement kinematic analysis, could reveal additional layers of motor-cognitive interaction in stroke recovery. Ultimately, this framework lays the groundwork for a truly comprehensive assessment of how USN manifests across the entire visuomotor cascade in a naturalistic setting.

## Abbreviations

AE: absolute error
AR: augmented reality
ATE: absolute trajectory error
FVE: free visual exploration
MGP: mean gaze position
Mocap: motion capture
RBL: right brain lesion
USN: unilateral spatial neglect
VR: virtual reality
